# A Novel Fixation Method of the Graft to the Fibular Head in Knee Lateral Collateral Ligament Reconstruction: Technical Note

**DOI:** 10.1055/s-0043-1768626

**Published:** 2024-04-10

**Authors:** Mesut Uluöz

**Affiliations:** 1 Departamento de Ortopedia e Traumatologia, Universidade de Ciências da Saúde, Hospital de Treinamento e Pesquisa da Cidade de Adana, Adana, Turquia

**Keywords:** anterior cruciate ligament reconstruction, collateral ligaments, joint instability, knee injuries, sports injury

## Abstract

The lateral collateral ligament (LCL) is the strongest lateral stabilizer of the knee. It provides support against varus stress and posterolateral rotation of the knee. Lateral collateral ligament injuries mostly occur together with anterior and/or posterior cruciate ligament injuries. While grades 1 and 2 injuries are treated conservatively since they are partial injuries, total ruptures, as in grade 3, require surgical treatment. In conventional LCL reconstruction methods, hamstring grafts are used, and bioscrews are used in bone-tendon fixation. Lateral collateral ligament reconstruction is usually performed as a component of multiple ligament surgery. Therefore, there is a need for a contralateral hamstring tendon or allograft. The present article aims to define a technique that does not require tendon grafts and bioscrews in fibular fixation.

## Introduction

Knee ligament injuries are among the injuries that orthopedists and traumatologists encounter quite often. Posterolateral and lateral ligament injuries account for 18 to 26% of significant knee ligament injuries. 1 Structures that support knee stability on the lateral side consist of the lateral collateral ligament (LCL), the fabelofibular ligament, the joint capsule, the popliteus tendon, the popliteofibular ligament, and the arcuate ligament. 2 Among these, the LCL is the primary stabilizer in preventing the varus angulation of the knee. Moreover, it supports the PCL in resisting external rotation and the ACL in resisting anterior translation. 3 If untreated, it may lead to permanent instability and pain in the knee. With increased varus gait, it may cause medial meniscus lesion and then medial osteoarthritis. Lateral complex injuries emerge with ACL/PCL injuries in many cases. In cases of LCL failure that is not noticed at the stage of diagnosis, the success of cruciate ligament reconstructions decreases over time. Many open, percutaneous, and endoscopic techniques have been described in LCL reconstruction. 4 Hamstring autografts are used in almost all these techniques. However, particularly in multiligament reconstructions, the need for a patellar tendon or allograft comes into prominence in addition to the hamstring autograft. 5 Donor site problems of the patient, who has already undergone surgical trauma, caused by taking the patellar tendon graft, and difficulties in reaching the allograft are obvious. A tensor fascia lata graft, which can be reached without making a significant incision in addition to the incision made in standard surgery, was used in the technique we describe.

In a study, a 7-mm tensor fascia lata graft was found to be significantly stronger than a 10-mm bone-patellar bone graft and a T. posterior graft. 6 In their study, Donahue et al. compared the T. anterior tendon, the T. posterior tendon, and hamstring tendon grafts. The authors reported that the hamstring graft was significantly weaker than the other two grafts. In the same study, they showed that the fascia lata graft became thicker as it distally approached the Gerdy tubercle. 7 In the technique we propose, we obtain a biomechanically quite strong graft since we take the fascia lata graft by starting from the place of adhesion to the Gerdy tubercle and proceeding proximally.

Moreover, bioscrews are used in fibular fixation in most of the techniques that are currently performed. Due to the metaphysis of the fibular head and the weakness of the cortex structure, a fracture may occur around the tunnel during screwing. 8 9 This risk is lower in the technique we describe. We aimed to describe a safe and easy technique to be used in isolated LCL or multiligament injuries.

## Indications

Lateral collateral ligament injuries are usually observed together with ACL and/or PCL injuries. 10 Grade 1 and 2 injuries are conservatively treated because there is no total rupture. We implement this method in grade 3, in which the ligament is totally ruptured.

## Surgical Technique

### Graft Harvesting and Preparation

**Fig. 1:**
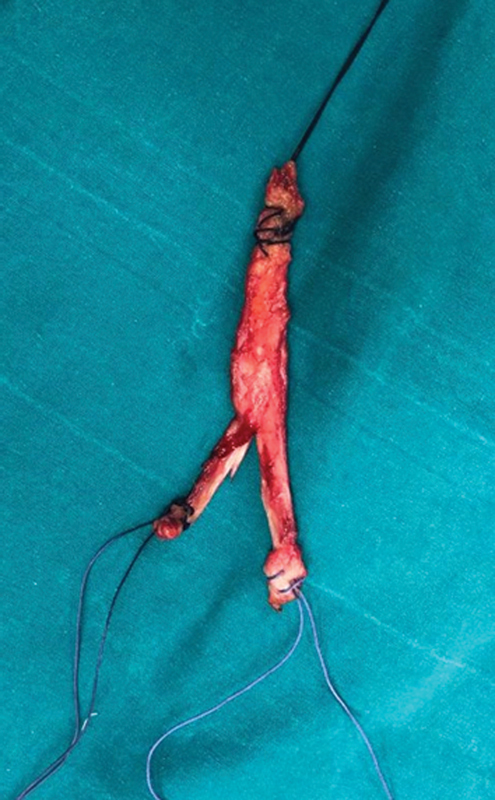
Image of the prepared graft.

The anesthetized patient is placed in the supine position. A tourniquet is placed around the proximal thigh, the leg is released down, and the tourniquet is inflated. The leg is disinfected and draped appropriately for asepsis. The fibular head and the lateral epicondyle are marked with a sterile pen. The plan for taking the tensor fascia lata graft is prepared. The fascia is reached by making an oblique longitudinal incision 2 cm anterior to the lateral condyle. Starting from the Gerdy tubercle and proceeding proximally, a graft with a thickness of 8 mm and a length of 10 cm is taken. It is put on a separate sterile table, and one end is longitudinally separated into two in the middle. The length of the separated legs should be longer than the anterior-posterior distance of the fibular head. It is prepared using nonabsorbable sutures (Ethibond No:2) via the Krackow suture technique with one end on one side and two ends on the other side ( Fig. 1 ). The graft thickness is measured, and the graft is wrapped in a sponge soaked with physiological saline.

### Fibular Tunnel and Fibular Fixation of the Graft

**Fig. 2 (A):**
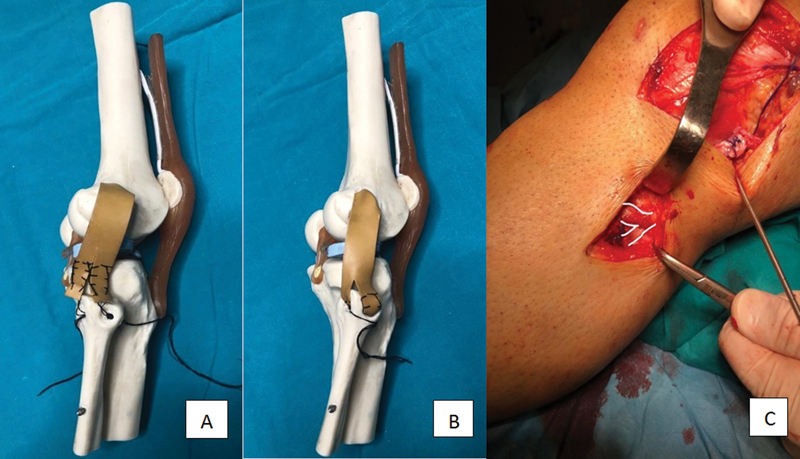
Drawing of the graft carried to the tunnels **(B)** Drawing of the fixation of the graft passed through the tunnels **(C)** Intraoperative image of fibular fixation.

A 5-cm vertical skin incision is made on the fibular head. The tunnel location is decided, and the guidewire is sent in the anteroposterior direction first. A tunnel is opened with a cannulated drill according to the graft thickness measured. At this stage, care should be taken against the risk of peroneal nerve injury. Before the distal graft is divided into two, its thickness should be measured. According to this measurement, the thickness of the tunnel at the head of the fibula is decided. The distal graft is divided into two longitudinally and sutured. Two looped carrier sutures are passed through the tunnel simultaneously. The length of the tunnel is measured to ensure that the separated leg length of the graft is longer than the tunnel. One of the graft legs is carried from anterior to posterior and the other from posterior to anterior with carrier sutures ( Fig. 2A ). Sutures are pulled from both sides, and the graft legs are seen to enter the tunnel. The sutures coming out of both sides are firmly tied on the fibula head to achieve fibular fixation ( Fig. 2B-C ).

### Femoral Tunnel and Femoral Fixation of the Graft

**Fig. 3 (A):**
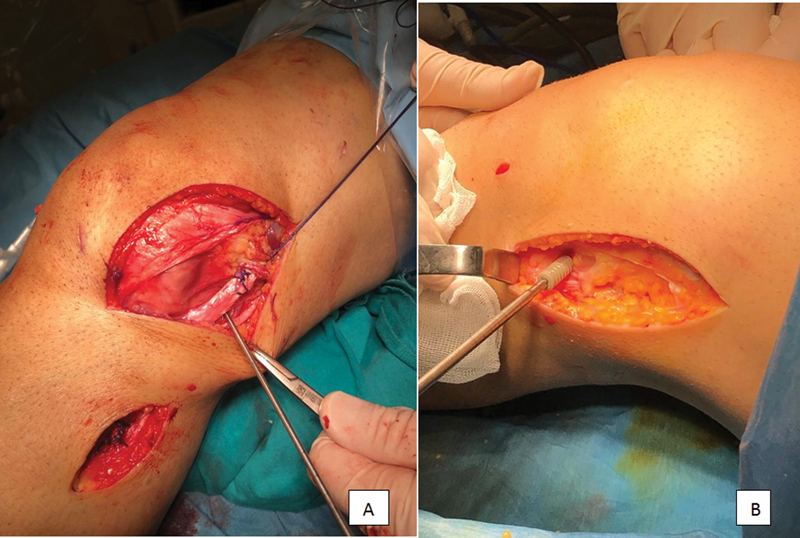
Measurement of the graft taken to the proximal side with a guidewire, calculation of the femoral tunnel length **(B)** Femoral fixation of tendon graft.

The lateral femoral epicondyle is found through the incision previously made to take a tensor fascia lata graft. The guidewire is proceeded toward the superomedial, and a scopy check is performed. The other end of the graft fixed to the fibula is carried from under the iliotibial band to the proximal incision by means of a clamp. The length of the graft, which will be stretched toward the guidewire and remain in the tunnel, is measured ( Fig. 3A ). The femoral tunnel is opened by a cannulated drill with an appropriate thickness for the graft. The tunnel is carved 10 mm longer than the length of the graft planned to be kept in the tunnel. The carrier suture is passed using the guidewire. Graft sutures are carried to the medial with carrier sutures. The sutures coming out of the medial are pulled strongly to help the graft enter the tunnel. It is fixed with a bioscrew while the knee is forced for valgus in 30° flexion ( Fig. 3B ). Upon checking with the varus stress test, the fascia lata defect is carefully closed.

### Postoperative Follow-up and Rehabilitation

**Fig. 4 (A):**
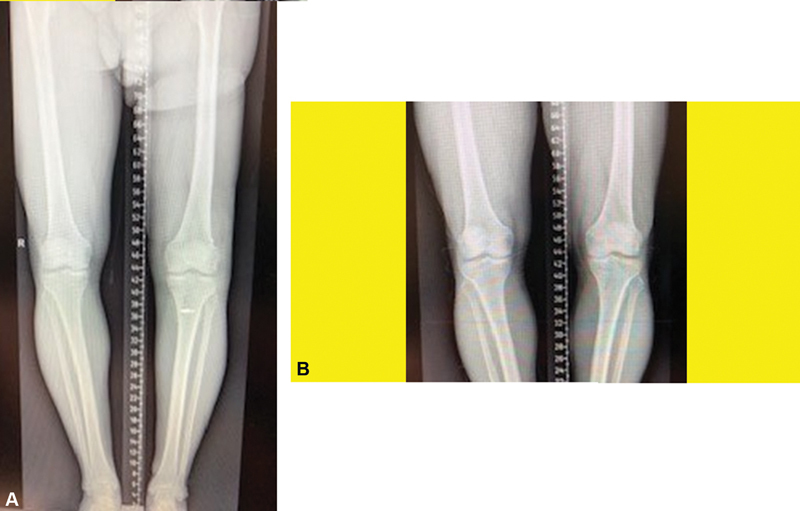
Preoperative orthorontronogram **(B)** Postoperative 6 ^th^ month orthorontronogram.

**Fig. 5 (A):**
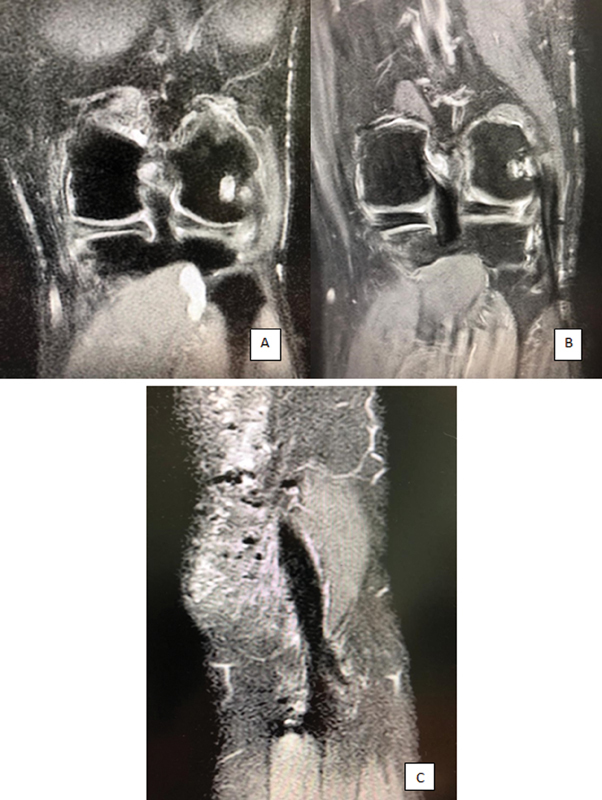
Preoperative magnetic resonance imaging (coronal) **(B)** Postoperative 6 ^th^ month magnetic resonance imaging (coronal) **(C)** Postoperative 6 ^th^ month magnetic resonance imaging (sagital).

In the rehabilitation of isolated LCL reconstruction, an angle-adjusted brace should be placed on the knee and fixed at 30°. In the first 2 weeks, aggressive patella mobilization and knee movement with range of movement (ROM) between 30° and 90° should be started. After 2 weeks, movement between 0° and 90° degrees is allowed, whereas no load is put on the knee until the 6 ^th^ week. The knee brace can be removed except for the periods when load is put on the knee. Between the 6 ^th^ and 12 ^th^ weeks, gradual loading and flexion tolerated up to 120 degrees are allowed. If there is no instability in the 6 ^th^ -month check-up, full movement without restriction is allowed. Moreover, if there are coperformed ACL/PCL reconstructions, they should also be considered during follow-up. Preoperative orthorontronogram shows varus deformity in the knee joint. This is an indication of lateral instability of the knee. It is seen that stability has been achieved in the standing x-ray at 6 months postoperatively ( Fig. 4 ). Lateral collateral ligament injury is seen in the preoperative magnetic resonance imaging (MRI) (coronal) ( Fig. 5A ). On the MRI images taken at the 6 ^th^ postoperative month, the reconstructed LCL is seen as intact ( Fig. 5B-C ).

## Discussion

The strongest aspects of the technique we have described are that tendon grafts are not needed and there is no need for bioscrews for fibular fixation. Many techniques have been described for LCL reconstruction. Tendon grafts are used in commonly applied techniques. 4 Considering that it is usually applied together with ACL or PCL reconstruction, there is a need for ipsilateral patellar tendon, contralateral hamstring tendons, or an allograft. There is no need for tendon grafts in the technique we have described. Since the grafted area is already in the area of the incision made for LCL reconstruction surgery, it does not cause any serious additional morbidity. In another technique, which was suggested to reduce the size of incision and the complication rate, the fibular-tibial tunnel was opened, and the graft was fixed from the medial of the tibia with the EndoButton. Thus, it was aimed to increase the strength of fibular fixation. However, it was emphasized that there was a risk of tunnel collision if ACL and PCL reconstructions were performed simultaneously. We think that our fibular fixation strength is satisfactory in the technique we have proposed. In our technique, since the graft has two legs in the distal, the leg in the anterior position resists varus stress by imitating the LCL. The leg in the posterior position provides partial support for the resistance to rotation, as in the Larson technique. In the technique we have described, there are fewer incisions than in conventional techniques, and no extra fixation material (bioscrew, EndoButton) is used for fibular fixation. There are studies stating that the most common intraoperative complication is a complete or incomplete fracture of the fibular head. 8 9 Especially the use of no bioscrews in fibular fixation considerably reduces the risk of fracture of the fibular head. This aspect is also an important advantage of our technique.

In multiligament injuries, it can be used together with the “Lemaire technique” if ALL reconstruction is to be added to the patient's operation. Two grafts of 8 mm can be taken from the tensor fascia lata, and the same procedure can be applied using the same tunnel in the femur.

The fixation method applied in this technique and the bioscrew method should be biomechanically compared. We are aware that this biomechanical study is imperative. Therefore, we also initiated the biomechanical study of this technique. So far, we have used this technique in nine of our patients. We have a clinical study plan by increasing the number of patients. The absence of biomechanical studies and the low number of patients are limitations of our study.

## Conclusion

In the present study, the tensor fascia lata graft is used, and screw fixation is not required for fibular fixation. The advantages and disadvantages of this technique compared with conventional methods are presented in Table 1 . This technique appears as an easy, safe, and less costly technique.

**Table 1:** Advantages and disadvantages of the technique described

Advantage	Disadvantage
It is a convenient method that can be applied in lateral instabilities in which rotation instability is not at the forefront.	It makes a limited contribution to rotational stability. In case of rotational instability, a relevant technique should be added.
There is no need for a significant additional incision during graft taking.	The incision is longer compared with percutaneous and endoscopic techniques.
There is no need for tendon grafts (allo/autograft).	
There is no need for implants for fibular fixation.	
Since no bioscrews are used, the risk of fibular head fracture is extremely low.	
It costs less than conventional methods.	
