# Supplementary material for: A Novel Fixation Method of the Graft to the Fibular Head in Knee Lateral Collateral Ligament Reconstruction: Technical Note
Source: Rev Bras Ortop (Sao Paulo). 2024 Apr 10;59(2):e307–12. doi: 10.1055/s-0043-1768626 (PMC11006509; doi:10.1055/s-0043-1768626)
Supplement: Related texts

# Research article: Um novo método de fixação do enxerto para a cabeça fibular na reconstrução do ligamento colateral lateral do joelho: Nota técnica

**Keywords:** instabilidade articular, lesões do esporte, ligamentos colaterais, reconstrução do ligamento cruzado anterior, traumatismos do joelho

## Abstract

O ligamento colateral lateral (LCL) é o estabilizador lateral mais forte do joelho. Ele fornece suporte contra o estresse varus e a rotação de posterolateral do joelho. As lesões de LCL ocorrem principalmente com lesões do ligamento cruzado anterior e/ou posterior. Enquanto as lesões grau 1 e 2 são tratadas de forma conservadora, uma vez que são lesões parciais, rupturas totais, como no grau 3, requerem tratamento cirúrgico. Utilizam-se enxertos isquiotibiais nos métodos convencionais de reconstrução da LCL e bioscrews na fixação do tendão ósseo. A reconstrução do LCL é geralmente realizada como um componente de cirurgia de ligamento múltiplo. Portanto, há necessidade de um tendão contralateral ou aloenxerto. O presente artigo tem como objetivo definir uma técnica que não exija enxertos tendíneos e bioscrews na fixação fibular.

## Introdução

Lesões no ligamento do joelho estão entre as lesões que ortopedistas e traumatologistas encontram com mais frequência. As lesões nos ligamentos posterolateral e lateral são responsáveis por de 18 a 26% das lesões significativas no ligamento do joelho. 1 As estruturas que apoiam a estabilidade do joelho na lateral consistem no ligamento colateral lateral (LCL), no ligamento fabelofibular, na cápsula articular, no tendão poplíteo, no ligamento popliteofibular e no ligamento arcuato. 2 Entre eles, o LCL é o estabilizador primário na prevenção da angulação em varo do joelho. Além disso, apoia o LCP na resistência à rotação externa e o LCA na resistência à translação anterior. 3 Lesões nestes ligamentos que não forem tratadas, podem resultar em instabilidade permanente e dor no joelho. O aumento da marcha em varo pode causar lesão no menisco medial e, em seguida, osteoartrite medial. Em muitos casos, lesões laterais complexas surgem com lesões de LCA/LCP. Nos casos de falha do LCL não notada na fase de diagnóstico, o sucesso das reconstruções do ligamento cruzado diminui com o tempo. Muitas técnicas abertas, percutâneas e endoscópicas foram descritas na reconstrução da LCL. 4 Autoenxertos tendíneos são usados em quase todas estas técnicas. No entanto, particularmente em reconstruções multiligamentares, a necessidade de um tendão patelar ou de um enxerto ganha destaque, além do autoenxerto do tendão. 5 Os problemas no local doador do paciente, que já sofreu trauma cirúrgico, causado pela extração do enxerto do tendão patelar, e as dificuldades em alcançar o aloenxerto são evidentes. Um enxerto de tensor da fascia lata, que pode ser alcançado sem fazer uma incisão significativa, além da incisão feita em cirurgia padrão, foi utilizado na técnica que descrevemos.

Em um estudo, verificou-se que um enxerto de tensor da fáscia lata de 7 mm foi significativamente mais forte do que um enxerto osseopatelar de 10 mm e um enxerto T. posterior. 6 Em seu estudo, Donahue et al. compararam o tendão T. anterior, o tendão T. posterior e os enxertos tendíneos. Os autores relataram que o enxerto do tendão foi significativamente mais fraco do que os outros dois enxertos. No mesmo estudo, eles mostraram que o enxerto de fascia lata ficou mais espesso à medida que se aproximava do tubérculo de Gerdy. 7 Na técnica que propomos, obtemos um enxerto bem forte biomecanicamente, uma vez que extraímos o enxerto de fascia lata, partindo do local de adesão ao tubérculo de Gerdy e procedendo proximalmente.

Além disso, os bioscrews são usados na fixação fibular na maioria das técnicas realizadas atualmente. Devido à metafise da cabeça fibular e à fraqueza da estrutura do córtex, uma fratura pode ocorrer ao redor do túnel durante o parafuso. 8 9 Este risco é menor na técnica que descrevemos. Nosso objetivo era descrever uma técnica segura e fácil de ser usada em lesões isoladas de LCL ou multiligamentares.

## Indicações

As lesões de LCL são geralmente observadas juntamente com lesões de LCA e/ou LCP. 10 As lesões de graus 1 e 2 são tratadas de forma conservadora porque não há ruptura total. Implementamos este método no grau 3, no qual o ligamento é totalmente rompido.

## Técnica Cirúrgica

## Extração e preparação de enxerto

O paciente anestesiado é colocado na posição supina. Um torniquete é colocado ao redor da coxa proximal, a perna é liberada para baixo, e o torniquete é inflado. A perna é desinfetada e coberta apropriadamente para assepsia. A cabeça fibular e o epicôndilo lateral são marcados com uma caneta estéril. O plano para retirar o enxerto do tensor fascia lata está preparado. A fascia é alcançada fazendo uma incisão longitudinal oblíqua anterior ao condílo lateral em 2 cm. Partindo do tubérculo de Gerdy e proximalmente, retira-se um enxerto com espessura de 8 mm e comprimento de 10 cm. Ele é colocado em uma mesa estéril separada, e uma extremidade é longitudinalmente separada no meio. O comprimento das partes separadas deve ser maior do que a distância anterior-posterior da cabeça fibular. Ele é preparado usando suturas não absorvíveis (Ethibond No:2) por meio da técnica de sutura de Cracóvia com uma extremidade de um lado e duas extremidades do outro ( Fig. 1 ). A espessura do enxerto é medida e o enxerto é envolto em uma esponja encharcada com soro fisiológico.

**Fig. 1:** Imagem do enxerto preparado.

## Túnel Fibular e Fixação Fibular do Enxerto

Uma incisão vertical da pele de 5 cm é feita na cabeça fibular. A localização do túnel é decidida e o fio-guia é enviado primeiramente na direção anteroposterior. Um túnel é aberto com uma broca canulada de acordo com a espessura do enxerto. Nesta fase, deve-se tomar cuidado com o risco de lesão peroneal do nervo. Antes que o enxerto distal seja dividido em dois, sua espessura deve ser medida. A espessura do túnel na cabeça da fíbula é decidida de acordo com esta medida. O enxerto distal é dividido longitudinalmente em dois e suturados. Duas suturas guia em loop são passadas pelo túnel simultaneamente. O comprimento do túnel é medido para garantir que o comprimento da perna separado do enxerto seja maior do que o túnel. Uma das partes do enxerto é transportada de anterior para posterior e outra de posterior para anterior com suturas guias ( Fig. 2A ). As suturas são puxadas de ambos os lados, e as partes do enxerto são colocadas para entrar no túnel. As suturas que saem de ambos os lados estão firmemente amarradas na cabeça da fíbula para alcançar a fixação fibular ( Fig. 2B-C ).

**Fig. 2 (A):** Desenho do enxerto transportado para os túneis **(B)** Desenho da fixação do enxerto que passou pelos túneis **(C)** Imagem intraoperatória de fixação fibular.

## Túnel Femoral e Fixação Femoral do Enxerto

O epicôndilo femoral lateral é encontrado por meio da incisão anteriormente feita para fazer um enxerto de tensor fascia lata. O fio-guia é direcionado para o superomedial e uma verificação de escopo é realizada. A outra extremidade do enxerto fixado à fíbula é transportada de baixo da faixa iliotibial para a incisão proximal por meio de um grampo. O comprimento do enxerto, que será estendido em direção ao fio-guia e permanecerá no túnel, é medido ( Fig. 3A ). O túnel femoral é aberto por uma broca canulada com uma espessura apropriada para o enxerto. O túnel é esculpido com 10 mm a mais do que o comprimento do enxerto planejado para ser mantido no túnel. A sutura guia é passada usando o fio-guia. As suturas de enxerto são levadas para o medial com suturas guias. As suturas que saem do medial são puxadas fortemente para ajudar o enxerto a entrar no túnel. Ele é fixado com um bioscrew enquanto o joelho é forçado para valgus em flexão de 30 graus ( Fig. 3B ). Ao verificar com o teste de estresse de varus, o defeito da fascia lata é fechado cuidadosamente.

**Fig. 3 (A):** Medição do enxerto levado para o lado proximal com um fio-guia, cálculo do comprimento do túnel femoral **(B)** Fixação femoral do enxerto tendinoso.

## Acompanhamento pós-operatório e reabilitação

Na reabilitação da reconstrução isolada da LCL, uma cinta ajustada a ângulo deve ser colocada no joelho e fixada a 30 graus. Nas duas primeiras semanas, deve-se iniciar a mobilização agressiva da patela e o movimento do joelho com amplitude de movimento (ADM) entre 30° e 90°. Após duas semanas, é permitido o movimento entre 0° e 90°, enquanto nenhuma carga é colocada no joelho até a 6ª semana. A joelheira pode ser removida, exceto pelos períodos em que a carga é colocada no joelho. Entre as 6ª e 12ª semanas, é permitida a carga gradual e a flexão tolerada até 120 graus. Se não houver instabilidade no seguimento do 6° mês, é permitido movimento completo sem restrição. Além disso, se houver reconstruções de LCA/LCP correalizadas, elas também devem ser consideradas durante o acompanhamento. O ortorontronograma pré-operatório mostra deformidade do varo na articulação do joelho. Isto é uma indicação de instabilidade lateral do joelho. Vê-se que a estabilidade foi alcançada no raio-x em pé no 6° mês pós-operatório ( Fig. 4 ). A lesão de LCL é vista na imagem de ressonância magnética (RM) pré-operatório (coronal) ( Fig. 5A ). Nas imagens de RM do 6° mês pós-operatório, o LCL reconstruído é visto como intacto ( Fig. 5B-C ).

**Fig. 4 (A):** Ortorontronograma pré-operatório **(B)** Ortorontronograma pós-operatório do 6° mês.

**Fig. 5 (A):** Ressonância magnética pré-operatória (coronal) **(B)** Ressonância magnética pós-operatório 6° mês (coronal) **(C)** Ressonância magnética pós-operatório 6° mês (sagital).

## Discussão

Os aspectos mais fortes da técnica que descrevemos são que enxertos tendinosos não são necessários e não há necessidade de bioscrews para fixação fibular. Muitas técnicas foram descritas para a reconstrução da LCL. Enxertos tendinosos são usados em técnicas comumente aplicadas. 4 Considerando que eles são geralmente aplicados juntamente com a reconstrução da LCA ou LCP, há necessidade de tendão patelar ipsilateral, tendões contralaterais ou aloenxertos. Não há necessidade de enxertos tendinosos na técnica que descrevemos. Como a área enxertada já está na área da incisão feita para cirurgia de reconstrução da LCL, não causa nenhuma morbidade adicional grave. Em outra técnica que foi sugerida para reduzir o tamanho da incisão e a taxa de complicações, o túnel fibular-tibial foi aberto e o enxerto foi fixado a partir da medial da tíbia com o EndoButton. Assim, buscou-se aumentar a força da fixação fibular. No entanto, enfatizou-se que havia risco de colisão de túneis se as reconstruções de LCA e LCP fossem realizadas simultaneamente. Achamos que nossa força de fixação fibular é satisfatória na técnica que propusemos. Uma vez que o enxerto tem duas partes no distal, a parte na posição anterior resiste ao estresse do varo imitando o LCL. A perna na posição posterior fornece suporte parcial para a resistência à rotação, como na técnica Larson. Na técnica que descrevemos, há menos incisões do que em técnicas convencionais e nenhum material de fixação extra (bioscrew, EndoButton) é usado para fixação fibular. Há estudos que afirmam que a complicação intraoperatória mais comum é uma fratura completa ou incompleta da cabeça fibular. 8 9 Especialmente o uso de bioscrews na fixação fibular reduz consideravelmente o risco de fratura da cabeça fibular. Este aspecto também é uma importante vantagem da nossa técnica.

Em lesões multiligamentares, a técnica pode ser usada juntamente com a "Lemaire" se a reconstrução do LAL for adicionada à cirurgia do paciente. Dois enxertos de 8 mm podem ser retirados do tensor fascia lata e o mesmo procedimento pode ser aplicado usando o mesmo túnel no fêmur.

O método de fixação aplicado nesta técnica e no método bioscrew devem ser biomecanicamente comparados. Estamos cientes de que este estudo biomecânico é imperativo. Por isso, também iniciamos o estudo biomecânico desta técnica. Até agora, usamos esta técnica em nove dos nossos pacientes. Temos um plano de estudo clínico aumentando o número de pacientes. A ausência de estudos biomecânicos e o baixo número de pacientes são limitações do nosso estudo.

## Conclusão

No presente estudo, é utilizado o enxerto de tensor fascia lata e a fixação do parafuso não é necessária para fixação fibular. As vantagens e desvantagens desta técnica em relação aos métodos convencionais são apresentadas na Tabela 1 . Ela aparece como uma técnica fácil, segura e menos cara.

**Tabela 1:** Vantagens e desvantagens da técnica descrita

Vantagem	Desvantagem
É um método conveniente que pode ser aplicado em instabilidades laterais nas quais a instabilidade de rotação não está na vanguarda.	Contribui limitadamente para a estabilidade rotacional. Em caso de instabilidade rotacional, deve-se adicionar uma técnica relevante.
Não há necessidade de uma incisão adicional significativa durante a extração do enxerto.	A incisão é mais longa em comparação com técnicas percutâneas e endoscópicas.
Não há necessidade de enxertos tendinosos (alo/autoenxerto).	
Não há necessidade de implantes para fixação fibular.	
Como não são utilizadas bioscrews, o risco de fratura na cabeça fibular é extremamente baixo.	
Custa menos do que os métodos convencionais.
